# Heat Treatment of Aluminum Alloys with the Natural Combination of Dopants

**DOI:** 10.3390/ma15165541

**Published:** 2022-08-12

**Authors:** Alisa Tsepeleva, Pavel Novák, Evdokim Kolesnichenko, Alena Michalcová, Zdeněk Kačenka, Jiří Kubásek

**Affiliations:** Department of Metals and Corrosion Engineering, University of Chemistry and Technology, Prague, Technická 5, 166 28 Prague 6, Czech Republic

**Keywords:** ferromanganese sea nodules, aluminum alloys, heat treatment

## Abstract

Aluminothermic reduction without the separation of individual metals is currently considered as a possible method for processing ferromanganese sea nodules and creating new alloys. In this study, the product of their reduction—a manganese-based polymetallic mixture—was added to pure aluminum, as a mixture of alloying elements in their natural ratios. After extrusion, two new aluminum alloys with a total percentage of metallic additives ranging from 1 to 6 percent were prepared. The possibilities of the precipitation strengthening of these aluminum alloys, especially those containing Mn, Fe, Si, Ni, and Cu, were investigated under a wide range of heat treatment conditions. After each tested combination of annealing and artificial aging temperatures, the phase composition and the microstructure changes were recorded by X-ray diffraction, optical, and scanning electron microscopy with EDS analysis. Under none of the tested heat treatment conditions is a significant hardening effect observed, even though the precipitate phases are observed by TEM. However, the changes in the morphology of the present intermetallic phases caused by the heat treatment are revealed, which highlights the further possible development of these multicomponent alloys.

## 1. Introduction

Currently, deep-sea nodules represent an alternative source of not only widely used industrial metals (Mn, Fe, Co, Ni, Li), but also of rare earth metals (Nd, Y, Ce, Sc, Eu). Polymetallic complexes form on the surface of ocean abyssopelagic zones, at depths of approximately 3500 to 6500 m [1,2]. Minerals based on manganese oxides and iron oxyhydroxides represent nodules’ morphology. Due to the high porosity of the structure (26–61%), the nodules’ surface is intensively subjected to redox reactions, contacts with microorganisms, and flow of pore fluids during formation, which ensures the adsorption and inclusion of metal ions (Ni^2+^, Cu^2+^, Co^2+^) into the matrix of complex and formation of coordination bonds [1,3,4]. The spatial dimensions usually range from 1 to 12 cm, with a mean mineral density 1.35 g/cm^3^. The most economically viable deposit is located in the Pacific Ocean between the so-called Clarion and Clipperton zones (CCZ), from where the raw material for our research was obtained [1,2,5].

The classical method of Fe–Mn nodules processing is the decomposition of a matrix in a reducing atmosphere, and the gradual extraction of target metals. In chemical metallurgy, this processing is carried out by two main methods: pyro- and hydrometallurgical methods [6]. A common characteristic of both processes is their multi-stage implementation. In case of pyrometallurgy, we talk about operations such as primary ore drying and calcination. A reduction in an electric arc furnace and separation of manganese-rich slag and iron triad metal-rich alloys usually follow. Next, the alloy is subjected to refining for the subsequent extraction of individual metals, while the slag is heat-treated to obtain a ferromanganese alloy [6,7]. In the case of hydrometallurgy, we considered several hours acid leaching, followed by the solvent extraction of metals; precipitation and electrolysis are also used. These operations require determination and the maintenance of specific environmental conditions such as atmospheric composition, temperature, and the pH of the solution to maximize the reduction in metals from compounds, in accordance with their E–pH diagrams [6,8,9]. Quantitatively incomplete reduction, a sometimes insufficient ratio of metals in the final alloy, and a presence of impurities are the main facts for the continuous improvement of both methods [7].

In order to simplify the technological and financial aspects of the question, a new method of deep-sea nodules processing—single-stage aluminothermic reduction without the separation of individual metals—was proposed and tested in recent research [10]. Compared to those described above, the new method is much less demanding in terms of energy, rapid, and environmentally friendly, due to the absence of emissions and non-usage of acidic solutions. A manganese-based alloy with a natural ratio of unseparated metals is a product of reduction. A natural combination of additives allows us to investigate this alloy for the presence of new properties [10]. Comparative analysis of samples obtained by reduction with different amounts of reducing agent (Al) shows consistently high hardness (around 800 HV1) and good wear resistance in contact with the thermally conductive material. With increasing aluminum concentration, a change in the manganese matrix phase, from cubic Mn_0,66_Ni_0,2_Si_0,16_ through the β–Mn phase to cubic α–Mn, is observed [10].

In this study, the polymetallic product of aluminothermic reduction was used as an additive to pure aluminum, in order to obtain new aluminum alloys with a certain concentration of metal additives. Manganese in the range from 1 to 2% is the main additive in the 3xxx series of aluminum alloys. It gives them ductility, and increases strength by stabilizing grain growth in the microstructure. These alloys are widely represented in the building, food, and automotive industries [11,12,13]. After the preparation of two types of new aluminum alloys, an attempt to find heat treatment conditions for precipitation hardening by dissolving some intermetallic phases was realized. The hypothesis was that the alloys are age-hardenable, because the reduced deep-sea nodules contain certain amounts of alloying elements, such as copper and nickel, which ensure the age hardenability. After each experiment, the hardness was measured, and the phase composition and microstructure were described.

## 2. Materials and Methods

Crushed deep-sea nodules powder, with the original chemical composition presented in Table 1, was subjected to aluminothermic reduction. The amount of reagent (Al) was calculated for the reduction of base metals (Mn, Fe, Cu, Ni, Co) from its oxides, and taken in the excess of 20 wt. %. The chemical composition of the reduction product is given in Table 2. The prepared manganese-based alloy, used as a polymetallic additive, was mixed up with pure aluminum using vacuum induction melting furnace in two percentages: 2 and 7 wt. % of reduced deep-sea nodules. After casting from the temperature of approx. 900 °C to the brass mold and hot extrusion (550 °C, extrusion ratio of 25:1) [14], billets of two new aluminum alloys, 6 mm in diameter and marked S1 and S2, were obtained. A summary of their chemical composition is given in Table 3.

X-ray fluorescence (XRF) analysis of all the above materials was performed using a spectrometer Axios (PANanalytical, Almelo, The Netherlands). Samples of both experimental alloys were subjected to differential thermal analysis (DTA) (thermoanalyzer TG-DTA Setsys Evolution (Setaram, Caluire-et-Cuire, France)) to determine the upper limit of heating temperature during solution annealing. Heat treatment of the experimental aluminum alloys was carried out in electric resistance heated muffle furnaces (Martínek, Ostrava, Czech Republic) with air atmosphere. Cold water was used as a medium for rapid cooling. Within the research, five heat treatment regimens were carried out under the conditions listed in Table 4.

Hardness measurement by the Vickers method was carried out periodically during processing: before the start, after cooling, and during artificial aging, in 0.5 h time intervals until the beginning of the overaging, which is connected to the coarsening and loss of the coherence of the precipitate. For this purpose, a microhardness tester Future Tech FM 700 (Future-Tech Corporation, Tokyo, Japan) with the load of 4.9 N (HV0.5) was used. After each experiment, the phase composition was determined by X-ray diffraction analysis (XRD) using a diffractometer X’Pert PRO (PANanalytical, Almelo, The Netherlands).

The microstructure of the alloys was investigated using an optical microscope Nikon ECLIPSE MA200 (Nikon Corporation, Tokyo, Japan) and scanning electron microscope (SEM) Tescan Vega 3 LMU (TESCAN, Brno, Czech Republic), equipped with an energy-dispersive (EDS) analyser (Oxford Instruments AZtec software, High Wycombe, UK). Before each microstructure analysis, the samples were subjected to conventional metallographic treatment procedures, comprised of grinding on silicon carbide abrasive papers (from P 240 to P 2500 grit) and polishing, using diamond paste D2 and suspension of colloidal silicon Eposil Non Dry. To visualize the microstructure, the samples were etched by Keller’s reagent of the composition: 2 mL HF (49–51%), 3 mL HCl (conc), 5 mL HNO_3_ (conc), and 190 mL distilled water [15].

The detailed analysis of the alloys’ microstructure, and the chemical composition of precipitates in particular, was realized by means of transmission electron microscope (TEM) Jeol 2200 FS (Tokyo, Japan) equipped with EDS spectrometer (Oxford Instruments AZtec software, High Wycombe, UK). TEM samples were prepared by mechanical grinding and ion-polishing in argon atmosphere, using Gatan PIPs system (Berwyn, PA, USA).

## 3. Results

### 3.1. Mechanical Properties

The melting process takes place in the temperature range 644–728 °C and 631–735 °C for the alloys S1 and S2, respectively. For these temperature ranges, the DTA curves show no further thermal effects. Based on the DTA results, the heat treatment regimens listed in Table 4 were applied.

**Figure 1:**
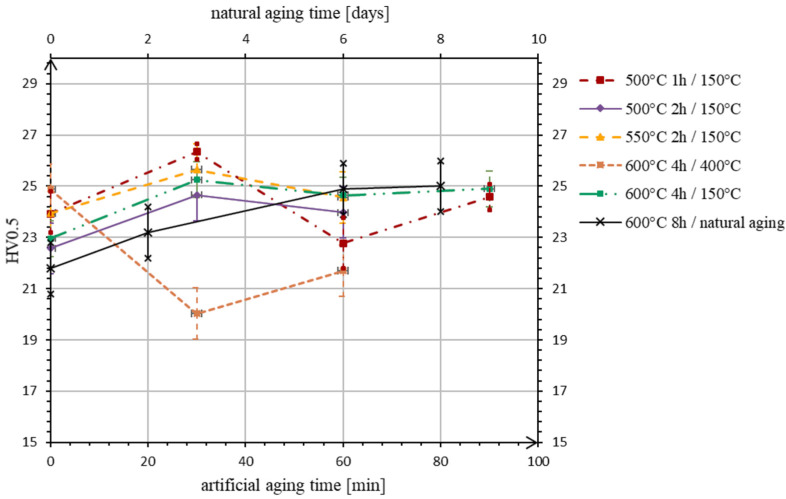
Change in hardness of alloy S1 during aging.

**Figure 2:**
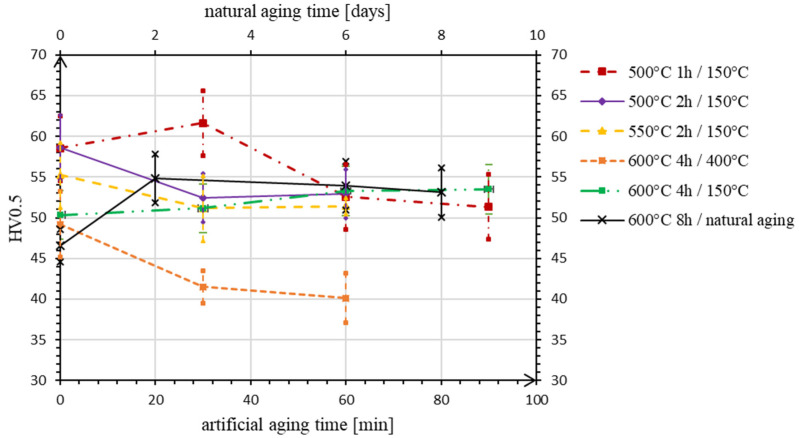
Change in hardness of alloy S2 during aging.

The mean values of hardness before the experiments were measured for alloy S1 27 ± 1 HV0.5, and for alloy S2 59 ± 3 HV0.5. According to the data measured during heat treatments, aging curves, i.e., the hardness vs. aging time plots, were constructed (Figure 1 and Figure 2), in which the first value (at time 0 min) is the hardness of the alloys after rapid cooling. As can be seen in Figure 1, for the alloy S1, the highest values of hardness during artificial aging are obtained after the first half-hour in all experiments, except in No. 3, where the sample is heated at a maximum temperature of 400 °C, and hardness decline is observed immediately. The highest hardness value of 26 HV0.5 is measured on the sample after the first experiment, but is still lower or comparable to the original one.

In case of the alloy S2 (Figure 2), the hardness increase is achieved in experiments No. 1 and 4, where the samples are heated at the same temperature of 150 °C, but after the different conditions of solution annealing. After holding the sample at 600 °C for 4 h (exp. No. 4) the artificial aging process is more moderate, with a steady slight increase in hardness. Despite this, the highest hardness of 62 HV0.5 is still measured within the first experiment. The value is higher than the value before the heat treatment procedure, but the increase is not statistically significant if we take into account the stated confidence interval. In the other experiments (No. 2 and 3), a decrease in hardness is observed promptly after the beginning of the aging process.

After comparing the data describing the progress of artificial aging, the experimental conditions in experiment No. 4 are chosen as a basis for the execution of natural aging—the solution annealing time is doubled, and the temperature is left unchanged as a maximum possible, taking into account the results of thermal analysis. In the case of the alloy S1, we observe a classic course of progress: the material hardness gradually increases and stabilizes on the sixth day of aging. The hardness value is equal to the result of the artificial aging in experiment No. 4, which is the highest value achieved after all heat treatment attempts 25 HV0.5.

In the case of alloy S2, after two days of aging at room temperature, the maximum hardness value of 55 HV0.5 is measured among all treatments at high temperatures (Figure 2). Further, the hardness decreases to artificial aging values in experiment No. 4.

### 3.2. Microstructure and Chemical Composition

Under the conditions of artificial aging No. 4, the heat treatment of both experimental alloys was carried out as close as possible to the classical process, i.e., a marked decrease in hardness after cooling and a slight increase during precipitation hardening were observed. Due to this, in order to illustratively compare and reveal possible changes, the results of analyses of alloys in their original state (after hot extrusion) and after heat treatments No. 4 and 5 (comparison of artificial and natural aging) are presented.

#### 3.2.1. Alloy S1

**Figure 3:**
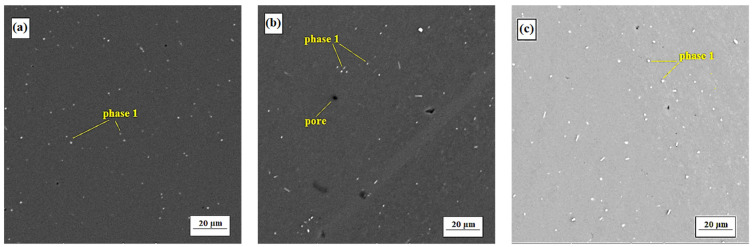
Microstructure of alloy S1 (SEM-BSE). (**a**) after hot extrusion. (**b**) after artificial aging No. 4. (**c**) after natural aging.

After each experiment of both artificial and natural aging, there was an overall increase in the amount of small-sized precipitates distributed in volume, which did not change by shape (Figure 3). However, the precipitate particles were significantly coarser than the coherent GP zones and the semi-coherent θ’ precipitate, which form during the aging process of aluminum alloys commonly. It can be highly expected that the interface between the matrix and these phases is incoherent and hence having the lower strengthening effect. At an initial stage of analysis, comparison of the chemical composition of precipitates by EDS made it possible to define them as one intermetallic phase (breakdown of the phase analysis is given below). In case of heat treatment with artificial aging, heating energy supported the mutual diffusion processes between matrix and phase. The matrix aluminum penetrated into the volume of phase, while the phase alloying elements dissolved in matrix (Table 5). In case of heat treatment with natural aging, diffusion of the alloying elements took place only during the solution annealing. After cooling, the temperature of aging was insufficient for mutual exchange of atoms by structural units, so the chemical composition of matrix and intermetallic phase remained virtually unchanged from the beginning of treatment (Table 5).

The differences in chemical composition can be probably explained by the small size of the particles of intermetallic phase. It causes that the local analysis of chemical composition in each particular case is highly distorted by the dominant signal of the surrounding matrix. The slight difference in iron and nickel content (Table 5) can be explained by the fact that, in comparison with natural aging, the heating energy during artificial aging created conditions for more active grain growth. Larger formed phases have been easily analyzed by EDS, being larger than the interaction volume. While in the smaller phases after natural aging any changes were not determined in comparison with the original state after extrusion (Table 5), which, however, does not guarantee their absence.

#### 3.2.2. Alloy S2

**Figure 4:**
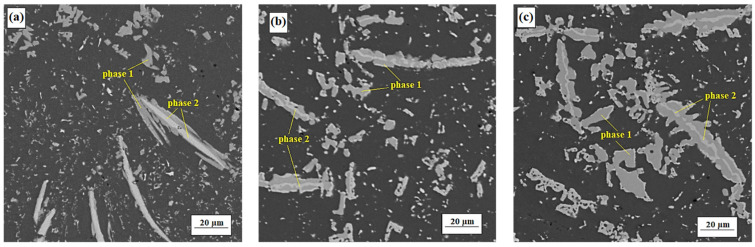
Microstructure of alloy S2 (SEM-BSE). (**a**) after hot extrusion. (**b**) after artificial aging No. 4. (**c**) after natural aging.

During heat treatment, on the contrary, the amount of particles-dispersoids was reduced due to their aggregation into larger agglomerates (Figure 4). The large needle-shaped intermetallic phases did not dissolve during the solution annealing, but there were partial changes in their morphology. The phases themselves, increasing in width, formed lateral branches of different sizes, depending probably either on the running of diffusion into matrix or on the presence of dispersoids in ambient volume.

Two intermetallic phases are clearly distinguished by their chemical composition (Table 6). Phase one with higher aluminum content, due to direct contact with the aluminum matrix, forms the outer shell of complex intermetallic formations (darker areas in SEM pictures, Figure 4). Phase two is characterized by a higher content of alloying elements (Mn, Si) and forms the inner core of intermetallic complexes (lighter areas in SEM pictures). Comparative analysis of the chemical composition showed that, regardless of the type of heat treatment, in all cases there is a general trend: mutual atom exchange between phases with the loss of some elements and the gain of others. This exchange can be illustrated by the change in the Mn/Fe ratio: in phase one was observed an increase from 2.9 to 5, while in phase two there was a decrease from 6.6 to 4.4 (5.9 in case of natural aging). As can be seen, in comparison with artificial aging, the reduced energy of natural aging has obviously led to a slowing down of diffusion processes.

### 3.3. Phase Composition

Each set of samples was subjected to X-ray diffraction analysis (XRD) after processing. The obtained diffraction patterns were evaluated and compared using HighScore Plus software (PANanalytical, Almelo, The Netherlands).

The common point of the prepared alloys is the aluminum solid solution matrix characterized by a cubic system (space group is Fm$\bar{3}$m) with a small content of diffused Mn (0.4–0.7 at. %) and Fe (0.1–0.2 at. %) atoms. Due to the slow diffusion processes during natural aging, matrix of the sample with initially higher percentage of additives (S2) after heat treatment also contained Si and Cu atoms that have penetrated it during the solution annealing and fixed after cooling.

**Figure 5:**
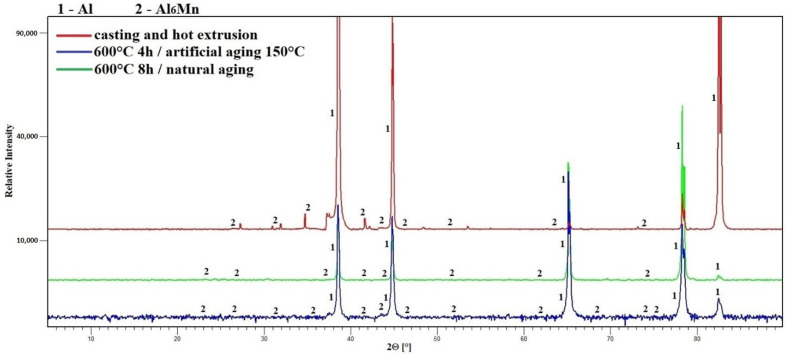
Phase composition of alloy S1 (XRD).

**Figure 6:**
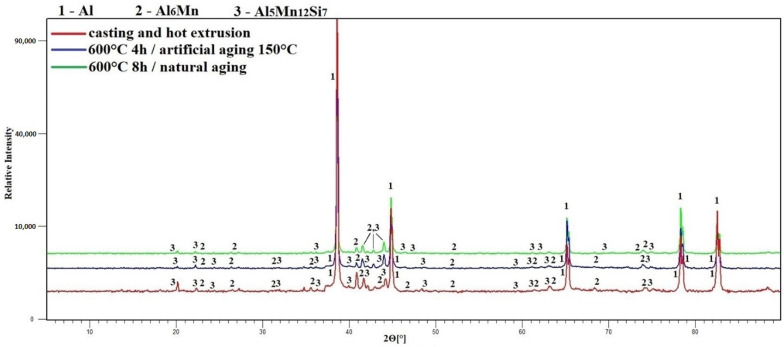
Phase composition of alloy S2 (XRD).

The next common structural unit of all alloys was phase one, which was defined as orthorhombic phase Al_6_ (Fe, Mn) (space group is Cmcm). In case of alloy S1 with a minimum content of alloying elements, this phase has formed in the aluminum matrix as a single stable. According to the diffraction data, it was already present after alloy preparation, probably changing only quantitatively after heat treatment (Figure 5). In case of alloy S2 phase Al_6_ (Fe, Mn) was also present in the matrix initially (Figure 6) and, as was already mentioned above, formed the outer shell of complex intermetallic structures. During the heat treatments, the phase was diffusively enriched by manganese losing iron at the same time. The result was an almost twofold increase of the ratio Mn/Fe. No difference between artificial and natural aging was found.

In addition to phase one, the diffraction data of the S2 alloy samples allows the establishment of phase two as cubic phase Al_5_Mn_12_Si_7_ (space group is Pm$\bar{3}$) (Figure 6). By assumption, after the formation of the main stable phase Al_6_Mn, metastable dispersoids of the Al_5_Mn_12_Si_7_ structure with a high Mn/Fe ratio are formed inside the orthorhombic phase during the hot extrusion of as-cast alloy. The inner core of the needle-shaped intermetallic complexes most likely represents the agglomeration of smaller dispersoids of different shapes. During the heat treatments, as a result of diffusion processes, the ratio of Mn/Fe is markedly reduced, due to the transit of iron into dispersoids and the replacement of manganese by it. The obtained data can be compared with the works [16,17,18], in which iron and manganese-containing aluminum alloys of the 6xxx series were investigated. It is reported that after several processing steps (homogenization annealing at 560 °C for 8 h, reheating, and extrusion), the formed small dispersoids have an ordered simple cubic (sc) α-AlMnSi structure, with a Pm$\bar{3}$ space group and a high Mn/Fe ratio (>1.6). Further, when the ratio decreases, a trend towards the formation of coarser particles with a body-centered cubic (bcc) α-AlFeSi structure (space group Im$\bar{3}$) is noticed [18]. In particular, Strobel et al. [16] found out that the growth of particles and transition in crystal structure may be affected by the diffusion of Fe atoms at increasing temperatures and time of homogenization annealing. In our case, the space group transition probably does not occur, because the critical Mn/Fe ratio (1.6) is not crossed at a decrease, although Yoo et al. [18] noted that this critical value may depend on the heat treatment conditions and change.

**Figure 7:**
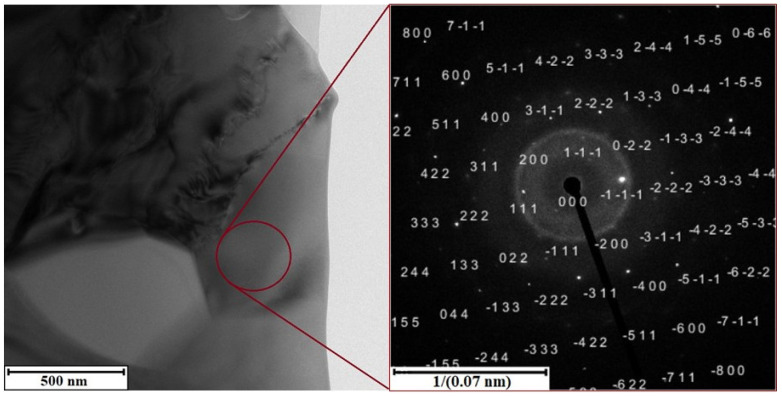
TEM micrograph of alloy S1 with electron diffraction of orthorhombic phase Al_6_(Fe, Mn).

**Figure 8:**
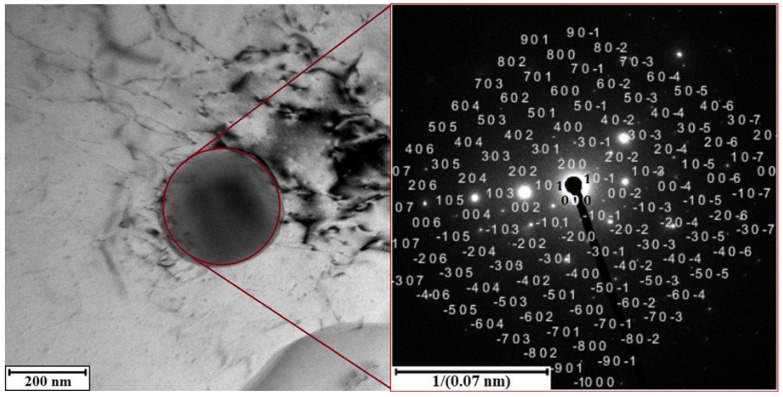
TEM micrograph of alloy S2 with electron diffraction of cubic phase Al_5_Mn_12_Si_7_.

**Figure 9:**
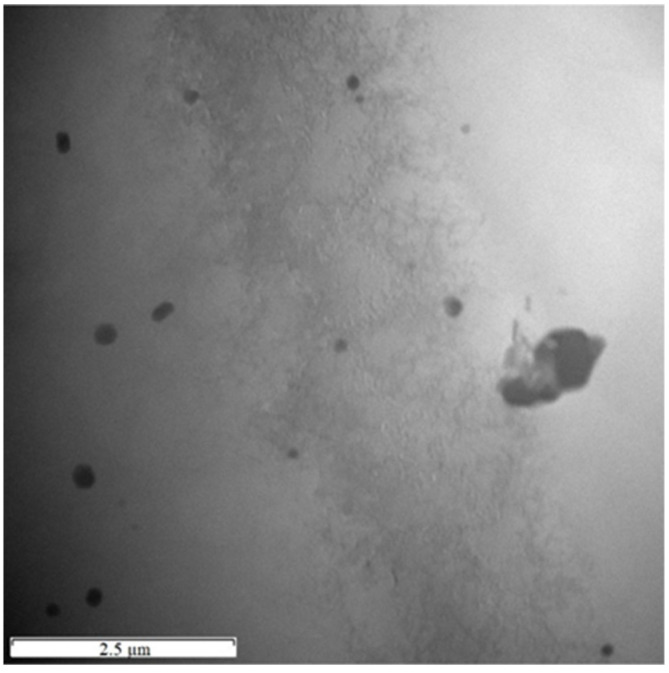
TEM micrograph showing fine intermetallics resulting from the heat treatment (exp. No. 4) in alloy S2.

Electron diffraction analysis cross-validates the results of X-ray diffraction. In the TEM images (Figure 7 and Figure 8) the selected areas for electron beam signal acquisition are marked. Using the software CrysTBox, the obtained diffraction patterns of both alloys clearly prove the structural presence of phase Al_6_ (Fe, Mn) (Figure 7). After manually entering the structural data of phase Al_5_Mn_12_Si_7_, the diffraction maxima corresponding to its crystallographic planes is identified in the alloy S2 (Figure 8). Fine precipitates of intermetallics are found in the S2 alloy after the heat treatment at 600 °C for 4 h and an artificial aging temperature of 150 °C (exp. No. 4) (Figure 9). However, it is not possible to identify them by SAED, due to their low volume fraction and small size.

## 4. Discussion

In terms of chemical composition (Table 3), only alloy S1 (0.31 wt. % Mn) is close to the wrought aluminum alloy of 3xxx series, according to the industrial classification. Matching can be performed with alloy 3102 (0.2 wt. % Mn). In the alloy S2 (4.23 wt. % Mn), manganese content is almost three times higher than the maximum value used in Al–Mn alloys as standard. Despite this, after preparation, our experimental alloys contain intermetallic phases characteristic for commercial 3xxx alloys such as orthorhombic Al_6_Mn and cubic α-Al(Mn, Fe)Si, whose distribution and precipitation behavior after heat treatments have been thoroughly investigated [19,20,21]. In addition, the small amount of copper (mainly in the S2 alloy) could also have a positive effect on the mechanical properties in response to thermal stress.

According to initial hardness, alloy S1, with a summary content of metal additives 1.16 wt. % (Mn, Fe, Si, Cu, and Ni), corresponds to wrought aluminum 1xxx alloys.

In general, the heat treatment process leads to the fragmentation of the intermetallic phase by the mutual diffusion of alloying elements and aluminum atoms in the solid matrix solution, and probably to the formation of new phase nuclei around these dispersed atoms. The volume distribution of precipitates in the matrix within all experiments results in a slight hardness increase. As can be seen from the comparison of experiments No. 2 and 4, increasing the solution annealing temperature by 100 °C and the heating time by 2 h has no significant effect. Experiment No. 3, on the other hand, shows that increasing the artificial aging temperature classically leads to an amplified expression of incoherence between the precipitates and matrix, which results in an immediate overaging (Figure 1). In the last experiment, No. 5, with natural aging, the maximum value of hardness is achieved, comparable with the results of artificial aging in experiment No. 4, but after a longer time (several days of aging in air at laboratory temperature).

Heat treatment of alloy S2, with a summary content of metal additives 7.47 wt. % (Mn, Fe, Si, Cu, and Ni), still does not lead to a significant change in hardness. By assumption, the highest value of hardness 62 HV0.5 during artificial aging No. 1 (Figure 2) is measured probably due to the initial insufficient hardness reduction after rapid cooling—the solution annealing conditions (500 °C, 1 h) are not enough to transform the microstructure, so the original hardness 59 HV0.5 does not change. Within other cases (exp. No. 2–5), a steady hardness decrease after cooling is observed according to a particular trend: the higher annealing temperature and time leads to the higher drop.

In the case of exp. No. 2 and 3, the incoherence between the large intermetallic phases and matrix immediately manifest at the beginning of artificial aging, followed by overaging. Only in exp. No. 4 does the combination of maximum possible annealing temperature, long exposure time, and moderate temperature of aging show a theoretically correct course of progress. To verify, these conditions are accentuated in the last experiment. Doubled annealing time and natural aging bring the alloy hardness to its maximum value (within our research), which, after stabilization, equals the artificial aging result.

The comparative analysis of phase composition of all samples does not reveal any change that is related to the heat treatment conditions. The following conditions can be considered satisfactory for both alloys at this time, namely: the highest temperature and time of solution annealing according to thermal analysis (in our case it is 600 °C and 4 h), and the lowest temperature of aging.

**Figure 10:**
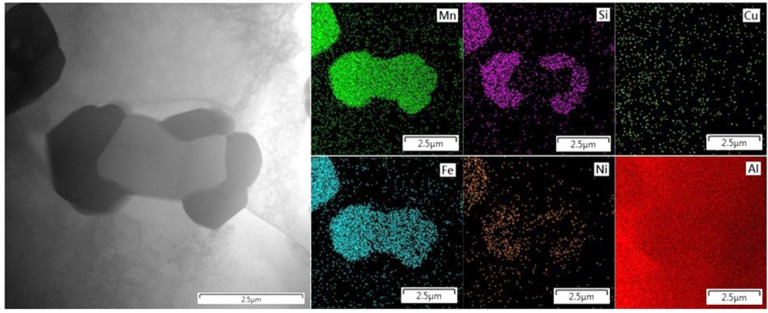
HAADF image (black and white left) and elemental mapping image of alloy S2 (STEM–EDS).

Detailed point analysis (STEM–EDS) shows the content of 82.9 ± 0.2 wt. % Al, 10.9 ± 0.1 wt. % Mn, 2.8 wt. % Fe, 2.7 wt. % Si, and 0.9 wt. % Ni in the revealed finer intermetallic particles. Based on several elemental maps (one of them is shown in Figure 10), it is assumed that the diffusion of the alloying elements occurs in the volume of grains, rather than along their boundaries. This is indirectly indicated by the uniform distribution of metal atoms without accumulation on the grain boundaries and, at the same time, by the very low concentration at a short distance from their main dislocation—the intermetallic phase Al_5_Mn_12_Si_7_. In the theory, the activation energy Q of grain boundary diffusion is several times less than the activation energy of volume diffusion [22,23,24], i.e., the first one runs more intensively with higher values of alloying elements’ atom concentrations at the endpoint of movement. Of course, the possibility of a combination of the two types of diffusion is not excluded.

**Figure 11:**
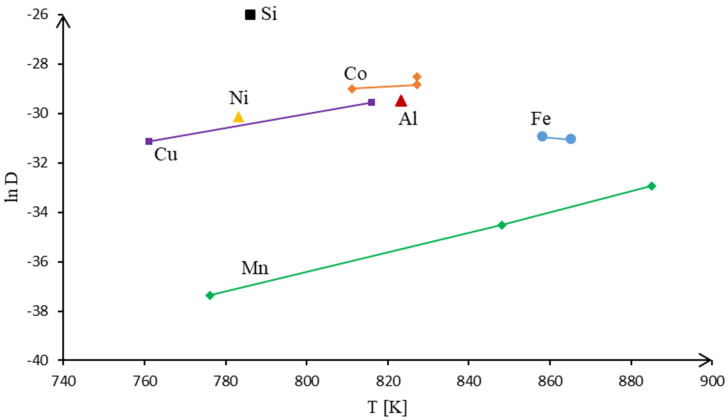
Comparative analysis of calculated self-diffusion coefficients of the main embedded metals.

Based on the assumption described above, the diffusion coefficients of individual alloying elements forming precipitates in aluminum were calculated, based on the constants for volume diffusion [24,25], using the Arrhenius-type equation, $D=D_{0}\exp\left(-\frac{Q}{\mathrm{RT}}\right)$, where $D_{0}$ is the pre-exponential coefficient [22,26] and R is the universal gas constant. The results of calculations are graphically presented in Figure 10. As can be seen, the mobility of the main (by mass) alloying elements, mainly Mn and Fe, during diffusion is much lower than that of the matrix metal. The more mobile alloying metals are either represented in the alloys in excessively low amounts (Cu), or concentrated in the volume of intermetallics (Si, Ni) (Figure 11). Based on this, we can speculate about the reason why the mechanical properties of 3xxx aluminum alloys do not change during heat treatment—manganese-rich intermetallics form a stable structure with a strong bonding of their constituent metals, while manganese itself shows practically no diffusive activity.

## 5. Conclusions

The main results can be summarized as follows:

- Two new wrought aluminum alloys were prepared, in which the product of an aluminothermic reduction of ferromanganese deep-sea nodules was used as the alloying mixture. The alloy samples were subjected to the set of heat treatments;
- After comparing the mechanical properties, phase, and chemical composition, slight changes in the microstructure are found, but it does not lead to a significant increase in hardness;
- None of the tested heat treatment conditions lead to a significant hardening, even though the precipitation is observed. It shows that the amount of copper, nickel, and other elements, which could ensure precipitation strengthening, is not high enough to form the suitable intermetallic phases in sufficient amounts;
- The alloying elements are bound in stable manganese-rich intermetallics, or in the aluminum-based solid–solution matrix;
- A qualitative description of the heat treatment conditions, which can ensure the correct processing, was given, and the physical parameters for quantitative determination of these conditions, such as the phase transformation on heating, were identified.

## Figures and Tables

**Table 1:** Chemical composition (XRF) of original deep-sea nodules (in wt. %) [10].

Element	Mn	Fe	Si	Al	Mg	Ca	Na	Cu	Ni	Ti	Zn	Co
wt. %	30.57	4.41	3.53	2.16	1.87	1.84	1.64	1.18	1.14	0.35	0.14	0.13

**Table 2:** Chemical composition (XRF) of reduced deep-sea nodules (in wt. %).

Element	Mn	Fe	Al	Si	Cu	Ni	Mo	Co	P
wt. %	57.71	15.36	9.31	8.00	3.93	4.08	0.21	0.54	0.49

**Table 3:** Chemical composition (XRF) of experimental aluminum alloys (in wt. %).

Element	Al	Mn	Fe	Si	Cu	Ni	Zn	P	Co	Mo
Alloy S1	98.70	0.31	0.07	0.71	0.04	0.03	0.01	0.01	-	-
Alloy S2	92.25	4.23	1.24	1.35	0.28	0.37	0.01	0.03	0.05	0.02

**Table 4:** Conditions of heat treatment of experimental aluminum alloys.

Experiment No	Solution Annealing		Temperature of Artificial Aging [°C]
	Temperature [°C]	Time [h]	
1	500	1	150
2	500	2	150
	550	2	
3	600	4	400
4	600	4	150
5	600	8	Natural aging

**Table 5:** Chemical composition of structural phases of alloy S1 (in at. %) (SEM-EDS).

	After Casting and Hot Extrusion		After Artificial Aging No. 4		After Natural Aging	
Element	Matrix	Phase 1 Al_6_(Fe, Mn)	Matrix	Phase 1 Al_6_(Fe, Mn)	Matrix	Phase 1 Al_6_(Fe, Mn)
Al	100	97.9	99.9	99.4	99.9	97
Mn		0.3	0.1	0.2	0.12	0.2
Fe		1.8		0.4	0.03	1.8
Ni						0.8

**Table 6:** Chemical composition of structural phases of alloy S2 (in at. %) (SEM-EDS).

	After Hot Extrusion			After Artificial Aging No. 4		
Element	Matrix	Phase 1 Al_6_(Fe, Mn)	Phase 2 Al_5_Mn_12_Si_7_	Matrix	Phase 1 Al_6_(Fe, Mn)	Phase 2 Al_5_Mn_12_Si_7_
Al	99.3	86.3	76.3	99.3	85.3	78.4
Mn	0.7	10.2	17.8	0.4	12.3	13.3
Fe		3.5	2.7	0.2	2.4	3
Si			3.2			4.8
		**After Natural Aging**				
**Element**	**Matrix**	**Phase 1** **Al_6_(Fe, Mn)**		**Phase 2** **Al_5_Mn_12_Si_7_**		
Al	99	85		78.2		
Mn	0.5	12.1		13.6		
Fe	0.1	2.3		2.3		
Si	0.3	0.4		5.2		
Cu	0.2	0.1		0.1		
Ni		0.1		0.5		

## Data Availability

The data are stored by the authors of the paper, not available publically.
